# Overview of Pharmacokinetics and Liver Toxicities of Radix Polygoni Multiflori

**DOI:** 10.3390/toxins12110729

**Published:** 2020-11-21

**Authors:** Dan Li, Mengbi Yang, Zhong Zuo

**Affiliations:** School of Pharmacy, The Chinese University of Hong Kong, Hong Kong 999077, China; 1155133238@link.cuhk.edu.hk (D.L.); yangmb1022@gmail.com (M.Y.)

**Keywords:** radix polygoni multiflori, herb induced liver injury, pharmacokinetics, mechanism, herb–drug/herb interaction

## Abstract

Radix Polygoni Multiflori (RPM), a traditional Chinese medicine, has been used as a tonic and an anti-aging remedy for centuries. However, its safe and effective application in clinical practice could be hindered by its liver injury potential and lack of investigations on its hepatotoxicity mechanism. Our current review aims to provide a comprehensive overview and a critical assessment of the absorption, distribution, metabolism, excretion of RPM, and their relationships with its induced liver injury. Based on the well-reported intrinsic liver toxicity of emodin, one of the major components in RPM, it is concluded that its plasma and liver concentrations could attribute to RPM induced liver injury via metabolic enzymes alteration, hepatocyte apoptosis, bile acids homeostasis disruption, and inflammatory damage. Co-administered 2,3,5,4′-tetrahydroxystilbene-2-*O*-*β*-D-glucopyranoside in RPM and other drugs/herbs could further aggravate the hepatotoxicity of emodin via enhancing its absorption and inhibiting its metabolism. To ensure the safe clinical use of RPM, a better understanding of the toxicokinetics and effect of its co-occurring components or other co-administered drugs/herbs on the pharmacokinetics of emodin is warranted.

## 1. Introduction

Radix Polygoni Multiflori (RPM) is the dry root of *Polygonum multiflorum* Thunb. (Fam. Polygonaceae). It could be used as raw material (raw RPM) or after steaming with black bean juice (processed RPM) in traditional Chinese medicine since the Tang dynasty with different indications [1]. According to Chinese Pharmacopeia, raw RPM at 3–6 g/person/day is mainly used for detoxification, eliminating carbuncle, preventing malaria, relaxing the bowel [1], while processed RPM at 3–12 g/person/day is used for nourishing the liver and kidney, supplementing the essence and blood, blackening hair, strengthening bones and muscles, eliminating dampness, and reducing lipids [1]. In addition to the clinical indications stated in Pharmacopeia, RPM and its major components, including 2,3,5,4′-tetrahydroxystilbene-2-*O*-*β*-D-glucopyranoside (TSG), emodin, emodin-8-*O*-*β*-D-glucopyranoside (EMG), and polysaccharides have also demonstrated pharmacological activities for anti-aging [2,3], immunomodulating [4,5], hepatoprotective [6,7], anticancer [8], and anti-inflammatory [9] effects, etc., in various preclinical studies.

Despite the wide use of RPM as a medicine or health supplement, an increasing number of hepatic adverse effect reports of RPM or proprietary Chinese medicinal products containing it have been constantly received since the 1990s in China and other countries [10,11,12,13,14,15,16]. Since the occurrence of hepatotoxicity cases associated with RPM has raised serious concerns regarding its safety in clinical practice, drug regulatory agencies of Canada, Australia, the United Kingdom (UK), and China have conducted monitoring of the usage of RPM [17,18,19,20]. To explore the potential hepatotoxicity mechanisms of RPM, many preclinical studies on the pharmacokinetic characteristics and liver injury mechanisms associated with RPM and its major constituents, including TSG, emodin, and physcion, have been performed. Besides hepatotoxicity, it was found that emodin, the major component of RPM, also has carcinogenic activity and kidney toxicity [21]. Although the botany, phytochemistry, quality evaluation, traditional uses, pharmacological research, and toxicology of RPM have been well-reviewed [22,23,24], there is no comprehensive information about the pharmacokinetic characteristics of RPM and mechanisms of its induced liver injury. To ensure the safe and effective application of RPM in clinics, we proposed a comprehensive overview and a critical assessment of the published data concerning the absorption, distribution, metabolism, excretion, and hepatotoxicity mechanisms of RPM components. Moreover, the potential relationship between the pharmacokinetics of RPM and its induced liver injury as well as the role of herb–drug/herb interaction in RPM induced liver injury is also discussed in our current review.

To achieve the above goals, the following databases were searched to identify relevant literatures in both English and Chinese: PubMed (from 1981 to September 2020) and China National Knowledge Infrastructure (CNKI, from 1988 to September 2020). Both Latin and Chinese pinyin terms, including “Polygoni Multiflori”, “*Polygonum mutliflorum*”, and “Heshouwu” were used as keywords to search the herb-related articles, and keywords including “2,3,5,4′-tetrahydroxystilbene-2-*O*-*β*-D-glucopyranoside”, “emodin”, and “physcion” were used for the search of compound-related articles. Exclusion criteria were as follows: (1) full text not available, (2) review articles on animal studies, (3) irrelevant in vitro studies using an herbal extract. In total, 54 articles, including 10 clinical studies, 35 animal studies, and 13 in vitro studies that contained information involving the pharmacokinetics and/or hepatic injury mechanisms of RPM or the major components, including TSG, emodin, and physcion, were identified in the current review, and the findings are highlighted as follows.

## 2. Chemical Constituents in RPM

**Figure 1:**
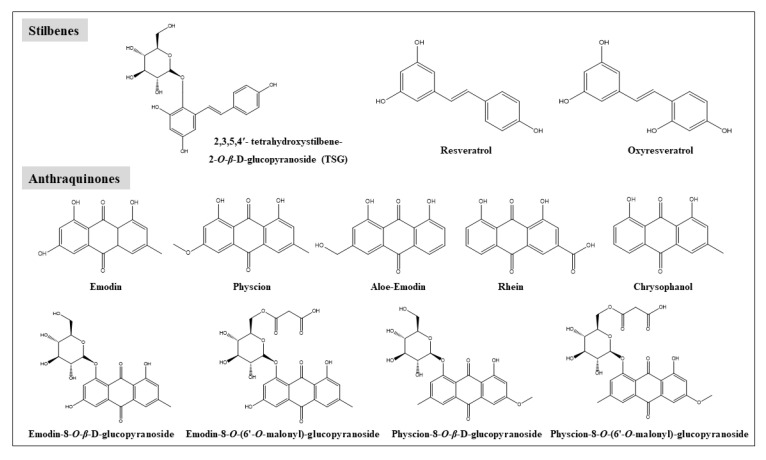
Chemical structures of the major components in Radix Polygoni Multiflori (RPM) extract.

Major chemical constituents in RPM include stilbenes, anthraquinones, flavonoids, and phenolic acids, etc. [25,26], with stilbenes and anthraquinones as the two major phytochemical groups for these components. As shown in Figure 1, among the stilbenes, including TSG, resveratrol, and oxyresveratrol, TSG is the most abundant. Among the major anthraquinones, including emodin, physcion, aloe-emodin, rhein, chrysophanol, EMG, emodin-8-*O*-(6′-*O*-malonyl)-glucopyranoside, physcion-8-*O*-*β*-D-glucopyranoside, and physcion-8-*O*-(6′-*O*-malonyl)-glucopyranoside. etc., emodin and EMG are the two most abundant [25,27,28]. Processing of RPM could decrease the contents of EMG and physcion-8-*O*-*β*-D-glucopyranoside and increase that of their corresponding aglycones, emodin, and physcion [28]. Chinese Pharmacopeia suggests that contents of TSG should not be less than 1.0% and 0.7% in the raw material and processed herb of RPM, respectively, and the combined contents of emodin and physcion should be greater than 0.1% in both raw and processed RPM [1]. The Hong Kong Standard of Material of Medica requires that the content of TSG should not be less than 2.2% in raw RPM [29].

## 3. Pharmacokinetics of RPM

### 3.1. Pharmacokinetic Studies of RPM Extract

So far, pharmacokinetics properties of the major components in RPM extract have primarily been investigated in rats. As shown in Table 1, TSG, emodin, EMG, aloe-emodin, physcion, oxyresveratrol, and rhein could be detected after oral administrations of RPM extracts (equivalent to 40 g raw RPM/kg) to rats. With the doses decreasing from 40 g/kg to 10 g/kg and 20 g/kg, oxyresveratrol and rhein became undetectable in plasma [25]. The pharmacokinetic parameters indicated that the absorption and elimination of these major components were generally fast with time to maximum plasma concentration (T_max_) less than 2 h for TSG, emodin, and physcion, and half-lives of these three compounds ranged from 0.18 to 8.37 h after a single dose of RPM extracts orally administrated to SD rats [25,27,30,31,32]. Since the area under the concentration-time curve (AUC) and peak concentration (C_max_) of TSG and emodin increased linearly along with the dose of RPM extract increasing from 10 g/kg (TSG: 327.9 mg/kg, emodin: 5.6 mg/kg) to 40 g/kg (TSG: 1312.0 mg/kg, emodin: 22.3 mg/kg), linear pharmacokinetics of TSG, and after single oral administration of RPM extracts in rats, were suggested [25].

The pharmacokinetics of TSG, emodin, and EMG after multiple dosing of RPM extracts to SD rats [33] found that the AUC and C_max_ values of TSG and emodin could significantly increase after 11 days of treatment of RPM extracts, which could be attributed to the change in metabolic enzymes after repeated RPM extracts administrations [34]. As for EMG, it was only detectable at a few time points after prolonged treatment of RPM, possibly due to its low content in RPM and low oral bioavailability in vivo.

### 3.2. Pharmacokinetic Properties of TSG, Emodin, and Physcion

Besides RPM extract, the pharmacokinetics of pure compounds of TSG, emodin, and physcion in beagle dogs and SD rats have also been studied and summarized in Table 2. Among these three compounds, only TSG and emodin have been investigated for their oral bioavailabilities. The absolute oral bioavailabilities of TSG were reported to be 24.2% and 36.5% for 50 mg/kg and 100 mg/kg in SD rats, respectively [35]. Oral administered 8 mg/kg of emodin resulted in 6%~9% bioavailability in SD rats. In addition, gender-specific pharmacokinetics of emodin was noticed with much higher C_max_/AUC, and a shorter half-life observed in male rats [36]. The T_max_ values among these major RPM components indicated their fast absorption, and comparison of the half-lives of these three compounds suggested the order of elimination rate as TSG > emodin > physcion [34,35,36,37,38,39,40]. The major characteristics of the absorption, distribution, metabolism, and elimination (ADME) for these compounds are highlighted below:

#### 3.2.1. Absorption

The intestinal absorption processes of TSG observed in the Caco-2 monolayer model, and the in situ intestinal perfusion model revealed its moderate intestinal permeability with an apparent permeability coefficient (P_app_) of TSG in the range of 1 to 10 × 10^−6^ cm/s [41]. Due to the significantly increased effective intestinal permeability (P_eff_) and absorption rate constant (K_a_) of TSG in the presence of verapamil hydrochloride, quinidine, and probenecid on the in situ intestinal perfusion model, transporters, including P-glycoprotein, and multidrug resistance-associated protein 2 (MRP2) were considered to be involved in the intestinal absorption of TSG [41]. After oral ingestion of TSG, the absorption rate of TSG was rather efficient with a T_max_ of 60 min and 15 min in beagle dogs [37] and rats [35], respectively.

The absorption behavior of emodin was explored in both the Caco-2 monolayer model (P_app_ A to B: 2 × 10^−6^ cm/s) and the rat in situ intestinal model (P_eff_: 1.2 × 10^−3^ cm/s) [42,43,44], and both suggested its moderate intestinal absorption. Besides, transporters, including Na^+^/glucose cotransporter (SGLT1), MRP2, and P-glycoprotein, were also involved in the efflux transport of emodin, leading to its poor oral bioavailability. It was found that phloridzin (SGLT1 inhibitor) reduced the absorption of emodin [45] and verapamil (P-glycoprotein inhibitor), and cyclosporine (MRP inhibitor) could increase the uptake of emodin in Caco-2 cells in a dose-dependent manner [43,45]. However, the MRP2 inhibitor (indomethacin), rather than verapamil hydrochloride, could significantly increase the K_a_ and P_eff_ of emodin in the rat in situ intestinal perfusion model [43], suggesting that MRP2 has more influence on the efflux transport of emodin than P-glycoprotein. The absorption rate of emodin was fast in both female and male rats with T_max_ less than 1 hour after ingesting 8 or 10 mg/kg of its pure compound [36,38].

Similar to TSG and emodin, physcion has a moderate intestinal permeability with P_eff_ values of (3.32 ± 1.50) × 10^−3^, (2.30 ± 1.57) × 10^−3^, (2.40 ± 0.58) × 10^−3^, (7.45± 3.30) × 10^−3^ cm/min in the duodenum, jejunum, ileum, and colon, respectively [46]. So far, there is no report on the transporter involved in the intestinal absorption of physcion yet. After oral administration of 26.4, 52.8, and 105.6 mg/kg physcion to rats, the absorption rate of physcion was fast with T_max_ of less than 1 h [40].

#### 3.2.2. Distribution

Among the major components in RPM, TSG demonstrated the quickest tissue distributions after its administrations in animals. It was detectable in tissues including heart, liver, spleen, lung, kidney, brain, small intestine, and stomach at 10 min after its oral administrations. Heart and kidney are the preferable tissues that TSG distributed to, followed by liver, lung, and stomach after 30 min post oral administration of 100 mg/kg TSG. Ten minutes after intravenous administration of TSG to rats, the liver was the major organ that TSG was preferably distributed to, followed by heart, lung, spleen, kidney, stomach, small intestine, brain, and testicles [35]. In summary, TSG showed an extensive and homogenous distribution into multiple tissues after both oral and intravenous administrations.

After oral administration of emodin, it was mainly distributed in the liver and kidney. Three hours after oral administration of 10 mg/kg emodin nanoformulation to rats, it could reach the peak concentrations in the major organs and distribute them in the order of liver, lung, kidney, heart, spleen, and brain [38]. In addition to these major organs, a sufficient amount of emodin was also found in mesenterium and adipose tissues after oral administrations of 10 mg [^14^C] emodin in rats [47]. Besides, a similar distribution in the liver and kidney in male rats, physcion was identified with gender-specific distribution due to no detectable amount in the tissues of female SD rats under the same experimental conditions [48].

#### 3.2.3. Metabolism

Recent preclinical studies have revealed that phase II metabolism is the major metabolic pathway of TSG. After incubating TSG with rat liver microsome for 60 min, only TSG glucuronide was determined [49]. After oral administration of TSG in rats, its glucuronidation metabolites were also identified as the major metabolites [35].

**Figure 2:**
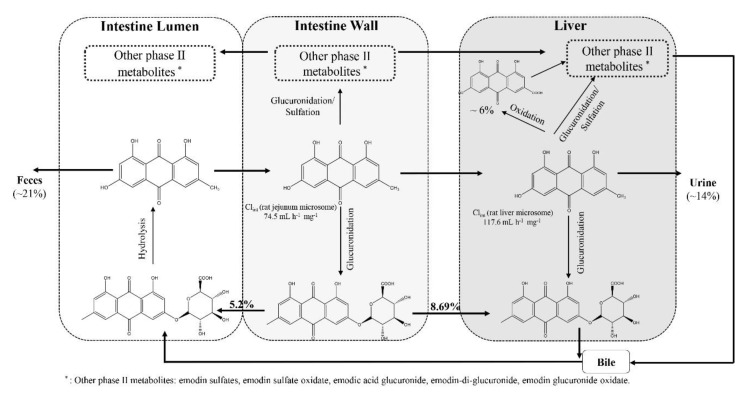
Illustrations of emodin metabolic pathways in rats.

Unlike TSG, emodin undergoes both phase I and phase II metabolism, with phase II metabolism to be the dominant one, as summarized in Figure 2. In rat liver microsome system, the oxidative metabolism of emodin was at least five times slower than its glucuronidation [50], and the total AUC of emodin glucuronide and emodin sulfate was extremely close to that of emodin glucuronide after oral administration of 20 mg/kg or 40 mg/kg emodin to rats [51]. Since the intrinsic clearance (CL_int_) of emodin in male rat jejunum (74.5 mL/(h·mg)) is very close to that of the liver (117.6 mL/(h·mg)) [50], much of the absorbed emodin was expected to be metabolized first in the intestine. After oral administration of emodin, it was absorbed across the intestine wall and converted into emodin-3-*O*-*β*-glucuronide (CL_int_ in rat jejunum microsome: 74.5 mL/(h·mg)) or other phase Ⅱ metabolites, such as emodin sulfates and other emodin glucuronides [50]. About 22.55% of the administered emodin appeared at the vascular side, including 12.01% free emodin, 8.69% emodin glucuronide, and 1.84% emodin sulfate [52]. In rat liver, emodin could be either oxidized into emodic acid (~6%), 2-hydroxyemodin, 4-hydroxyemodin, ω-hydroxyemodin, 3-carbomethoxy-6-methoxy-1,8-dihydroxyanthraquinone, and physcion [47,53], mainly by cytochrome P450 (CYP) 1A2 [54] or undergo phase II metabolism to form emodin glucuronides and emodin sulfates with emodin-3-*O*-*β*-glucuronide as the major metabolite [50,51]. The extensive metabolism via glucuronidation in rats may be one of the major reasons for the poor oral bioavailability of emodin in rats, which was reported to be 9.28% and 6.54% for male and female rats, respectively [36].

Similar to emodin, both phase Ⅰ and phase Ⅱ metabolites were identified for physcion. After incubating physcion with liver microsomes, the oxidation and demethylation products of physcion were found [55]. After oral treatment of physcion to rats, besides oxidative metabolites of physcion, physcion N-acetylcysteine conjugates, physcion sulfate, and physcion glucuronide were also detected. Moreover, recombinant human CYP1A2, 2C19, and 2B6 were demonstrated to be the primary enzymes mediating the hydroxylation of physcion [55].

#### 3.2.4. Elimination

Among the three major RPM components, TSG eliminated the fastest with no detectable drug in rat tissues at 1 h and 3 h after its intravenous and oral administrations, respectively [35]. Bile excretion of TSG peaked at 2 h after intravenous administration, with cumulative excretion of TSG and TSG monoglucuronides at 0.1% and 5.8% of the dosage at 24 h, respectively. The urinary and fecal cumulative excretion of unchanged TSG was 0.007% and 0.063% 24 h post-dosing, respectively. The quick elimination in rat tissues, low level of TSG in feces/urine, and high bile excretion of TSG monoglucuronides suggested its extensive biotransformation in the liver [35].

Similar to TSG, emodin is mainly excreted to bile, feces, and urine. After oral administration of 10 mg [^14^C] emodin to rats, 49%, 45.7%, and 6.9% of the dose was excreted to bile, feces, and urine in its parent or metabolites form [47]. Among the excreted emodin in bile, conjugated emodin and emodic acid were much higher than their non-conjugated forms, further confirming the phase II metabolism in the liver as the major metabolic pathway.

Physcion was found to be mainly excreted as unchanged form via feces, with 13%~21% recovered in feces at 72 h after oral administration of 18.7 mg/kg [48]. The urine excretion of physcion was rather limited, with less than 0.2% of the total dose found during the same period. So far, there is no report on the bile excretion of physcion.

#### 3.2.5. Effect of Co-Occurring Ingredients in RPM on the Pharmacokinetics of Emodin

In addition to the above-mentioned ADME of each individual component in RPM, there are a number of studies that reported the potential interaction between the co-occurring ingredients in RPM. After comparing the pharmacokinetic parameters of TSG and emodin after oral administration of RPM extracts (Table 1) versus that obtained from their pure components (Table 2) in SD rats, it was noticed that the half-life values of TSG and emodin after oral administration of RPM extracts (0.5~2 h for TSG, 1.5~3 h for emodin) are similar to that from their pure components (0.5 h for TSG, 3 h for emodin). However, the C_max_ and AUC values of emodin after oral administration of RPM extracts (dose of emodin: 11.17 mg/kg; AUC: 683.0 ± 268.9 ng h/mL, C_max_: 224.5 ± 131.1 ng/mL) were higher than that from pure emodin (10 mg/kg, AUC: 420.3 ± 48.1 ng h/mL, C_max_: 74.9 ± 17.4 ng/mL) to SD rats, suggesting that systemic exposure of emodin could be affected by other co-occurring ingredients in RPM extract. It was found that the presence of TSG could significantly increase the C_max_ and AUC values of emodin via inhibiting its metabolism [34,56]. Moreover, the in vitro study also confirmed that TSG could increase the absorption of emodin via inhibiting its MRP mediated transport in Caco-2 cells and UDP glycosyltransferase-(UGT) mediated glucuronidation in human liver microsomes in a dose-dependent manner [57]. In addition to the influence of TSG, it was reported that the co-occurring anthraquinones components, including aloe-emodin, rhein, chrysophanol, and physcion, may lead to a decrease in emodin AUC in the cerebral ischemia-reperfusion model rats [58]. Overall, since the contents of these components are much lower than TSG in RPM [1,25], TSG may have the most significant influence on the change in emodin pharmacokinetics, which warrants further experimental verification.

In summary, the ADME characteristics of the major components in RPM, including TSG, emodin, and physcion, were well studied in rats and beagle dogs with fast absorption, and the elimination of TSG was faster than emodin followed by physcion. The transporters, such as SLTC1, P-glycoprotein, and MRP2, were involved in the absorption of TSG and emodin with glucuronidation as their major metabolic pathway. Additionally, TSG could increase the systemic exposure of emodin via increasing its absorption and inhibiting its metabolism in a dose-dependent manner. However, there is no information about the biodistribution of the major components of RPM after its extract treatment, which can offer a better understanding of the toxicity of RPM, especially hepatotoxicity.

## 4. Hepatotoxicity

### 4.1. Case Reports on Liver Injury of RPM

So far, there are several retrospective analysis studies investigating the clinical cases on RPM induced liver injury [59,60,61]. According to these studies, the common reasons for consuming RPM products included treating grey hair, hair loss, using it as a health supplement, or for the treatment of hypertension, coronary heart disease, hyperlipidemia, etc. In addition to proprietary products of RPM, decoction pieces processed with water, alcohol, or ground into powder were commonly used for oral administration in clinics. For all the patients from the above-mentioned case reports, the onset time for liver toxicity ranged from 1 to 240 days with a median of 30 days after oral administrations of RPM at doses ranging from 1 g/person/day to 100 g/person/day.

According to the Roussel Uclaf Causality Assessment Method, based on the type of damaged target cells, liver injury can be classified into three types, including hepatocyte liver injury, cholestatic liver injury, and their mixture type [62]. Most of the liver injuries induced by RPM were diagnosed as hepatocellular injury followed by mixed liver injury and cholestatic liver injury with jaundice, fatigue, anorexia as the major symptoms of RPM induced liver injury. Although RPM can induce liver injury in different degrees and even lead to death, the majority of RPM associated liver damage was found to be reversible after discontinuing RPM products and conservative care [59,60,61].

### 4.2. Mechanistic Studies on Liver Injury Induced by RPM Extract and Its Major Components

Since hepatic adverse effect reports on RPM had raised much concern for its safe use in clinics, a series of studies have been conducted to investigate the mechanisms of RPM associated liver injury. The findings are summarized in Table 3 with the major mechanisms highlighted below.

#### 4.2.1. Metabolic Enzymes Alteration and Genetic Polymorphism

It was found that altered metabolic enzymes, such as CYP and UGT, were proved to contribute to RPM induced liver injury. The protein expression of drug metabolic enzymes, including CYP2A, CYP3A4, CYP2C19, CYP2E1, UGT1A1, and UGT1A8, was inhibited while the ALT and AST increased after oral administration of RPM extracts to rats [31]. In CYP1A2 or CYP2E1 inhibitors-treated rats, RPM could significantly increase the level of serum transaminases ALT and AST and induce moderate liver injury [63]. In addition, according to the clinical studies, the liver injury induced by RPM may be related to the polymorphism of CYP. CYP1A2, which account for 13% of total CYP enzymes in human [64], exhibits genetic polymorphism in the population, and CYP1A2*1C frequency (46.5%) in RPM induced liver injury Chinese patients was found to be significantly higher than that in healthy volunteers (27.9%) [65]. As the major absorbable component of RPM, emodin (greater than 6% of the dose) could undergo phase Ⅰ metabolism with CYP1A2 as the major metabolic enzyme in rats [54]. Thus, the altered CYP1A2 mediated metabolism of emodin may attribute to RPM induced liver injury. In addition to *CYP1A2* polymorphism in humans, it was found that RPM induced liver injury might be a type of immune-mediated idiosyncratic liver injury, and the frequency of the HLA-B*35:01 allele was much higher in RPM induced liver injury patients (45.4%) than healthy Han Chinese population (2.7%) [66].

Overall, the altered metabolic enzymes, including CYP and UGT, as well as HLA-B*35:01 allele, are considered to be high-risk factors for RPM induced liver injury.

#### 4.2.2. Hepatocytes Apoptosis

Apoptosis is defined morphologically on the basis of cellular rounding up, cytoplasmic shrinkage, chromatin condensation, and nuclear fragmentation [67]. Effector caspase activation is required for the acquisition of this morphology. As the most numerous cell type in the liver, the apoptosis of hepatocytes is prominent in liver injury [67]. Apoptosis shares common cell death machinery, including death receptor-dependent and mitochondria-dependent pathways [68]. As the major anthraquinone in RPM, emodin could induce liver damage via the mitochondrial pathway [69,70,71]. Yang et al. found that emodin could induce mitochondrial apoptosis and lead to liver injury with protein expression of caspase-9, caspase-3, and cytochrome c (Cyt c) increasing after treating the rats with emodin (1500 mg/kg) for one week [69]. Similar results were observed in the L02 cell line; that 50 μM emodin could affect oxidative phosphorylation pathways by inhibiting the function of the mitochondrial respiratory chain complexes, leading to mitochondrial damage and hepatocyte apoptosis in vitro [70]. In addition, emodin at 20–80 μM was found to block cell cycle progression and generate reactive oxygen species in HepaRG cells, leading to abrogated mitochondrial membrane potential and cell apoptosis via mitochondrial apoptosis pathway [71].

#### 4.2.3. Disruption of Bile Acids Homeostasis

Currently, bile acids have been demonstrated to play essential roles in drug-induced liver injury [72]. According to the clinical case reports about RPM induced liver injury, besides the hepatocellular injury induced by RPM, many cases of RPM associated liver injury were classified as cholestatic liver injury, suggesting a correlation between bile acids and RPM induced liver injury. The preclinical investigations in cell lines, mice, and rats indicated that RPM and its major anthraquinones could alter the disposition of bile acids at different degrees via regulating bile acid synthesis or transport [73,74,75,76,77,78].

RPM could alter the biosynthesis of bile acids. In mice, after oral administration of 1.275 and 3.825 g/kg RPM extract of processed RPM for 7 days, the bile acids levels in hepatocytes decreased, followed by the downregulation in the protein expression of CYP7A1, the key enzyme involved in bile acids synthesis [77]. After consecutively administrating 30 and 60 g/kg RPM extracts to rats for 28 days or 20 g/kg RPM extracts for 90 days, the protein expression of CYP7A1 was upregulated [73,74]. Such discrepancy in CYP7A1 regulation could be due to different animal species, different doses and duration of RPM treatment, which warrants further verification.

Besides influencing the biosynthesis of bile acids, RPM could also disturb the bile acid homeostasis via regulating the expression of bile acid transporters. The mRNA expression of sodium taurocholate cotransporting polypeptide (*Ntcp*), the major uptake transporter of bile acids, could be upregulated after one-week of oral administrations of 3.825 g/kg RPM extract to mice [77] and downregulated after oral administration of 20 g/kg RPM extracts for 3 to 7 weeks in rats [75]. Besides, the mRNA/protein expression levels of bile salt export pump (BSEP/*Bsep*) [74,75,77] and Mrp2/3 [74], the major efflux transporters for bile acids, were elevated after oral RPM extract treatment in mice (3.825 mg/kg for 7 days) [77] or rats (20–60 g/kg for up to 7 weeks) [74,75]. Moreover, mRNA and protein expression levels of farnesoid X receptor (FXR), which controls bile synthesis and transport, were inhibited after oral administration of 30 g/kg or 60 g/kg RPM extract for 28 days in rats [74]. Since the disruption of bile acids synthesis and transport, the balance of the bile acid pool could be disrupted. Therefore, glycochenodeoxycholic acid (GCDCA) in bile [76], hyodeoxycholic acid (HDCA) in serum [75,76], as well as tauro-β-muricholic acid (TβMCA) in urine [75] were suggested to be potential biomarkers for RPM induced liver injury in rats.

Furthermore, the in vitro study revealed that anthraquinones from RPM, including emodin, chrysophenol, and physcion, could alter the disposition of bile acids in sandwich cultured rat hepatocytes. Anthraquinones, including emodin, physcion, and chrysophanol, could significantly increase the total bile acids in the cells and bile duct at 25 μM and inhibit the basolateral efflux of bile acids at 50 μM [78]. Additionally, emodin and physcion at 50 μM could significantly inhibit the function of MRP2/3 and BSEP as well as regulating the mRNA expression of bile acids synthesis enzymes, transporters, including *Cyp7a1*, *Cyp27a1*, *Cyp8b1*, *Ntcp*, *Mrp2/3/4*, and *Bsep,* in sandwich cultured rat hepatocytes [78]. Since physcion and chrysophanol were almost undetectable after oral administration of RPM extracts, emodin, the major in vivo detectable component from RPM, could be considered as the main component contributing to the disruption of bile acids induced by RPM.

In summary, RPM extract could disturb the bile acid pool via regulating bile acid synthesis enzyme expression or affecting the function or expression of bile acid transporters. Several bile acids, such as GCDCA, HDCA, and TβMCA, were suggested to be potential biomarkers for RPM induced liver injury, which offers a foundation for the safe use of RPM in the clinic. Emodin, the major bioavailable anthraquinone in RPM, plays an important role in RPM induced bile acids homeostasis.

#### 4.2.4. Inflammatory Damage

Besides the mechanisms mentioned above, the inflammation response has an important effect on RPM induced liver injury. It was also found that emodin could induce inflammatory liver damage in vivo and in vitro. In lipopolysaccharide (LPS) treated rats, which was considered as idiosyncratic liver injury model, emodin at doses ranging from 20 to 80 mg/kg could significantly increase the level of plasma proinflammatory cytokines, such as TNF-α, IL-1β, and IL-6, as well as the level of AST and ALT [79]. Additionally, emodin could significantly increase the level of p-NF-κB and IL-6, which induce inflammatory damage in the L02 cell line in a dose-dependent manner [80].

### 4.3. Correlations between Pharmacokinetics of RPM and Its Induced Liver Injury

Similar to western drugs, RPM could exhibit therapeutic windows with toxicities identified at higher doses [81]. It was reported that RPM extract could attenuate liver cirrhosis induced by dimethylnitrosamine in mice at the dose of 20–100 mg/kg/day (equal to 0.093–0.465 g raw RPM/kg/day), while such therapeutic effect decreased and toxic effects were observed with the dose increasing to 500 mg/kg/day (equal to 2.326 g raw RPM/kg/day) [7]. A similar trend was also observed for emodin in rats with liver protection from CCl_4_-induced fibrogenesis after its oral administration at 40 mg/kg/day [82] and liver damage induced after its oral administration at 1500 mg/kg [69]. Moreover, Ma et al. indicated that the gradual increased in vivo exposure of emodin after oral administration of RPM extract (20 g raw RPM/kg) for 21 days might contribute to the RPM-induced hepatic lesions [33]. Therefore, it is speculated that the therapeutic and toxic effects of RPM could be correlated with the dose and in vivo level of emodin.

It is noticed that the maximum concentration of emodin in the rat plasma ranged from 61.29 ng/mL to 348.10 ng/mL after oral administration of RPM extract at doses ranging from 6 g/kg to 40 g/kg. Such concentration is far below 20 μM (or 54 μg/mL), the minimum concentration of emodin observed in the in vitro liver toxicity study in human L02 and HepaRG cells [70,71] and rat sandwich cultured hepatocyte [78]. Shi et al. reported a much higher concentration of emodin in the liver ( 940.12 ng/g) than that in the plasma (120.98 ng/ml) after oral administration of 10 mg/kg emodin loaded nanoemulsion in rats [38]. Although its human liver concentration remains unknown, emodin is expected to have a higher accumulation in the liver than in plasma, leading to potential liver toxicity.

In addition to the above-mentioned liver toxicity of emodin itself, the co-occurring components in RPM could also affect its in vivo levels leading to enhanced liver toxicity. Although both CYP1A2 and UGT1A8 were involved in the metabolism of emodin in rats, UGT mediated phase II metabolism is the dominant metabolic pathway of emodin. After consecutively treating with TSG (117 mg/kg) for 7 days, a decrease in the mRNA expression of *Ugt1a8* in rat liver and intestine led to increased C_max_ and AUC of emodin in rats [34], and the metabolism of emodin could be inhibited by TSG in the human liver microsome in a dose-dependent manner [57]. Moreover, the absorption of emodin could be increased in the Caco-2 cell in the presence of TSG. Such increased systemic exposure of emodin by TSG may further contribute to the RPM induced liver injury. According to the existing Pharmacopeia, only the lower limits for the contents of TSG and emodin in RPM were required. However, the impact of RPM with different contents of TSG on the in vivo concentrations of emodin and its related liver damage is not clear so far. Therefore, the relevant upper limits of the content of TSG and emodin in RPM, and the relationship with its induced liver toxicity need further clarification.

Toxicokinetics is usually adopted to determine the relationship between the systemic exposure of a compound and its toxicity in animals and humans. To achieve the toxicokinetics of herbal medicines, such as RPM, we need to determine the exposure of its major bioavailable components in blood and major organs and the relationship with its induced liver toxicity. Since there is no information about the concentrations of the major components of RPM in the liver after oral administration of its extract, detailed biodistributions (especially liver concentrations) of RPM major components in preclinical animal studies should first be obtained to better understand the in vivo levels of these components, including TSG and emodin, and how they correlate with the RPM induced liver injury.

### 4.4. Role of Herb–Drug/Herb Interactions in RPM Induced Liver Injury

Herb–drug/herb interactions are of great concern when patients concomitantly take drugs and herbs, especially taking herbal and western medicines at the same time. Since emodin was the major component contributing to liver toxicity of RPM, the interaction of emodin in RPM with other drugs/herbs could be critical to the safe use of RPM in the clinic.

It was noted that emodin induced hepatotoxicity at 150mg/kg could be further enhanced by probenecid (100mg/kg) due to increased systemic exposure of emodin resulted from its inhibition on UGTs and MRP2 in rats [83]. In addition, piperine, the bioactive compound of *Piper nigrum* L. and *Piper longum* L., could significantly increase the AUC and C_max_ of emodin via the inhibition of its glucuronidation [84]. Therefore, people should pay more attention to hepatotoxicity when they take emodin-containing herbal medicine together with drugs/herbs that could inhibit the expression or activity of UGT or MRP2. On the other hand, the herb–herb interaction may attenuate the RPM induced liver injury. A recent study found that combined use of Poria and RPM could significantly ameliorate the RPM-induced liver injury and systemic inflammation in LPS treated rats [85]. Since emodin could also induce liver injury in LPS treated rats with significantly increased proinflammatory cytokines [71], the above-mentioned detoxification effects of Poria could be related to its influence on emodin leading to a reduction in corresponding inflammatory cytokines, which warrants further verification.

## 5. Conclusions

In summary, plasma pharmacokinetic profiles of RPM and its major components have been investigated in various preclinical models, while the possible mechanisms of its induced liver injury have also been explored in the clinic as well as different preclinical models. Based on the evidenced liver toxicity of emodin, it was suggested that emodin was the major component attributed to RPM liver injury, and the co-occurring ingredient TSG could increase the exposure of emodin via inhibition of its phase II metabolism, leading to enhanced liver toxicity via hepatocyte apoptosis, disturbing bile acids homeostasis, and inflammatory damage. Besides, other major co-occurring anthraquinones components, including aloe-emodin, rhein, chrysophanol, and physcion, as well as herb–drug/herb interaction with RPM, also play important roles in its induced liver injury.

To further understand the impact of emodin in vivo levels on the RPM induced liver injury, its biodistributions in major organs, including plasma and liver, after oral administration of RPM extract should be evaluated to establish its toxicokinetics. In addition, the effect of contents of co-occurring components in RPM or other co-administered detoxification herbs on the in vivo levels of emodin and related liver toxicity is also worth further exploration. Based on the established toxicokinetics of emodin in animals, physiological toxicokinetic models could be adopted to describe and predict the behavior of emodin in humans, as suggested before [86]. Thus, clinical monitoring of its level in biomatrix could serve as an approach for the prevention and/or early diagnosis of RPM-induced liver injury in future clinical practice.

## Figures and Tables

**Table 1:** Plasma pharmacokinetic parameters of the major components in Radix Polygoni Multiflori (RPM) after oral administrations of its extract to SD rats.

Dose of RPM Extract (Equivalent Dose of Raw/Processed RPM) and Its Major Components	Pharmacokinetics Parameters	TSG	EMG	Emodin	Aloe emodin	Physcion	Oxyresveratrol	Rhein	Ref.
1.7 g/kg (10 g/kg of raw RPM) TSG: 327.9 mg/kg EMG: 2.9 mg/kg Emodin: 5.6 mg/kg Aloe Emodin: 1.1 mg/kg Oxyresverol: 0.08 mg/kg Physcion: 0.7 mg/kg Rhein: 0.05 mg/kg	T_max_ (h)	0.3 ± 0.1	0.2 ± 0.0	0.2 ± 0.2	0.2 ± 0.0	0.2 ± 0.1	ND	ND	[25]
	C_max_ (ng/mL)	74.4 ± 18.5	65.1 ± 58.3	152.4 ± 36.6	29.6 ± 7.4	11.6 ± 5.5	ND	ND	
	AUC_0→__∞_ (ng h/mL)	195.8 ± 151.2	95.3 ± 94.9	233.5 ± 120.5	137.9 ± 105.3	35.2 ± 21.9	ND	ND	
	T_1/2_ (h)	NA	NA	NA	NA	NA	NA	NA	
3.4 g/kg (20 g/kg of raw RPM) TSG: 655.8 mg/kg EMG: 5.7 mg/kg Emodin: 11.2 mg/kg Aloe Emodin: 2.2 mg/kg Oxyresverol: 0.2 mg/kg Physcion: 1.4 mg/kg Rhein: 0.1 mg/kg	T_max_ (h)	0.2 ± 0.0	0.2 ± 0.0	0.3 ± 0.1	0.4 ± 0.1	0.3 ± 0.2	ND	ND	
	C_max_ (ng/mL)	189.2 ± 46.7	115.1 ± 38.9	224.5 ± 131.1	40.7 ± 23.0	30.8 ± 11.0	ND	ND	
	AUC_0→__∞_ (ng h/mL)	350.4 ± 321.6	371.1 ± 340.6	683.0 ± 268.9	281.7 ± 203.5	203.3 ± 130.4	ND	ND	
	T_1/2_ (h)	NA	NA	NA	NA	NA	NA	NA	
6.8 g/kg (40 g/kg of raw RPM) TSG: 1312 mg/kg EMG: 11.4 mg/kg Emodin: 22.3 mg/kg Aloe Emodin: 4.4 mg/kg Oxyresverol: 0.3 mg/kg Physcion: 2.8 mg/kg Rhein: 0.2 mg/kg	T_max_ (h)	0.2 ± 0.0	0.2 ± 0.0	0.2 ± 0.0	0.2 ± 0.0	0.6 ± 0.4	0.2 ± 0.0	0.5 ± 0.2	
	C_max_ (ng/mL)	784.5 ± 543.9	160.3 ± 44.3	348.1 ± 131.5	106.2 ± 33.4	95.8 ± 51.6	0.6 ± 0.8	0.4 ± 0.2	
	AUC_0→__∞_ (ng h/mL)	2019.0 ± 431.9	492.1 ± 143.4	1042.2 ± 589.0	485.7 ± 151.0	438.1 ± 163.0	7.7 ± 13.1	3.1 ± 4.1	
	T_1/2_ (h)	NA	NA	NA	NA	ND	NA	NA	
NA (36 g/kg of raw RPM) TSG: 1170 mg/kg EMG: 31.4 mg/kg Emodin: 14.8 mg/kg Aloe Emodin: 13.7 mg/kg Oxyresverol: NA Physcion: 15.5 mg/kg Rhein: 8.8 mg/kg	T_max_ (h)	0.3 ± 0.1	0.3± 0.1	0.2 ± 0.1	0.2 ± 0.1	ND	NA	0.5 ± 0.1	[30]
	C_max_ (ng/mL)	1743.0 ± 401.0	101.0 ± 47.4	175.0 ± 33.8	11.3 ± 3.1	ND	NA	1.1 ± 0.2	
	AUC_0→__∞_ (ng h/mL)	1871.0 ± 581.0	83.7 ± 32.3	801.0 ± 233.0	8.5 ± 3.4	ND	NA	2.3 ± 0.5	
	T_1/2_ (h)	6.0 ± 2.62	3.9 ± 2.5	8.4 ± 4.2	3.4 ± 1.4	ND	NA	1.2 ± 0.4	
3.3 g/kg (19.19 g/kg of raw RPM) TSG: 78.8 mg/kg Emodin: 5.6 mg/kg	T_max_ (h)	0.5 ± 0.2	NA	0.2 ± 0.0	NA	NA	NA	NA	[27]
	C_max_ (ng/mL)	884.0 ± 146.0	NA	89.9 ± 13.6					
	AUC_0→__∞_ (ng h/mL)	3292.0 ± 707.0	NA	1842.0 ± 425.0					
	T_1/2_ (h)	1.1 ± 0.5	NA	2.8 ± 1.6					
1.7 g/kg (18.00 g/kg of processed RPM) TSG: 48.8 mg/kg Emodin: 12.6 mg/kg	T_max_ (h)	0.4 ± 0.1	NA	0.2 ± 0.0					
	C_max_ (ng/mL)	491.6 ± 179.7	NA	61.3 ± 9.2					
	AUC_0→__∞_ (ng h/mL)	1137.0 ± 401.6	NA	879.9 ± 195.0					
	T_1/2_ (h)	0.3 ± 0.0	NA	1.7 ± 0.6					
NA (6 g/kg of raw RPM) TSG: 212.2 mg/kg Emodin: 4.9 mg/kg EMG: 22.0 mg/kg	T_max_ (h)	0.2 ± 0.1	0.3 ± 0.1	1.6 ± 3.1					[31]
	C_max_ (ng/mL)	69.6 ± 51.8	21.5 ± 25.6	86.7 ± 19.7					
	AUC_0→__∞ _(ng h/mL)	90.2 ± 35.8	14.3 ± 15.9	506.3 ± 61.6					
	T_1/2_ (h)	2.2 ± 1.6	0.2 ± 0.1	5.0 ± 1.7					
NA (10 g/kg of raw RPM) No content report for TSG, emodin and EMG	T_max_ (h)	0.7 ± 0.1	1.0 ± 0.6	0.5 ± 0.3					[32]
	C_max_ (ng/mL)	240.2 ± 114.0	204.4 ± 85.9	76.7 ± 13.2					
	AUC_0→__∞ _(ng h/mL)	373.6 ± 142.7	489.7 ± 129.7	395.2 ± 208.3					
	T_1/2_ (h)	1.6 ± 0.6	1.7 ± 0.5	6.5 ± 1.4					

ND: not detectable; NA: not available.

**Table 2:** Preclinical plasma pharmacokinetic parameters of major RPM components after oral administrations of their pure compounds.

Compounds	Species	Dose, Route of Administrations	C_max_ (μg/mL)	T_max_ (h)	AUC_0→t_ (μg h/mL)	AUC_0→∞_ (μg h/mL)	T_1/2α_ (h)	T_1/2β_ (h)	Ref.
TSG	Beagle dogs	0.52 g/kg, p.o.	0.83 ± 0.04	1.00 ± 0.00	1.53 ± 0.07	2.04 ± 0.02	0.20 ± 0.02	0.56 ± 0.05	[37]
		0.78 g/kg, p.o.	1.16 ± 0.06	1.00 ± 0.00	2.30 ± 0.06	3.00 ± 0.19	0.10 ± 0.02	0.60 ± 0.03	
		1.04 g/kg, p.o.	2.17 ± 0.23	1.00 ± 0.00	3.60 ± 0.02	4.59 ± 0.35	0.14 ± 0.02	0.64 ± 0.15	
	SD rats	10 mg/kg, i.v.	22.80 ± 2.60	-	5.10 ± 0.33	5.84 ± 0.19	NA	NA	[35]
		20 mg/kg, i.v.	64.20 ± 3.60	-	11.01 ± 0.58	12.23 ± 0.98	NA	NA	
		50 mg/kg, p.o.	5.70 ± 1.60	0.25 ± 0.02	5.99 ± 0.59	7.09 ± 1.87	NA	NA	
		100 mg/kg, p.o.	21.90 ± 2.50	0.24 ± 0.02	20.70 ± 0.64	21.29 ± 0.63	NA	NA	
Emodin	SD rats	4 mg/kg, i.v.	5.83 ± 2.34	-	7.18 ± 1.84	NA	NA	1.38 ± 0.59	[36]
		8 mg/kg, p.o.	0.21 ± 0.09	0.30 ± 0.11	1.33 ± 0.53	NA	NA	6.42 ± 1.72	
		10 mg/kg, p.o.	0.08 ± 0.02	0.75 ± 0.00	0.39 ± 0.04	0.42 ± 0.05	NA	2.98 ± 0.71	[38]
		20 mg/kg, p.o.	6.04 ± 1.14	NA	13.18 ± 2.99	13.28 ± 3.00	NA	1.22 ± 0.29	[39]
		82.4 mg/kg, p.o.	0.10 ± 0.01	NA	1.26 ± 0.08	1.30 ± 0.02	4.56 ± 0.76	NA	[34]
Physcion	SD rats	26.4 mg/kg, p.o.	0.29 ± 0.12	1.00 ± 0.76	45.84 ± 36.00	NA	NA	13.25 ± 5.60	[40]
		52.8 mg/kg, p.o	0.41 ± 0.15	1.00 ± 0.42	47.52 ± 33.60	NA	NA	14.23 ± 11.00	
		105.6 mg/kg, p.o	0.49 ± 0.17	0.75 ± 0.56	78.70 ± 31.20	NA	NA	10.97 ± 6.60	

NA: not available.

**Table 3:** Summary of reported clinical and preclinical liver injury mechanisms of RPM and its components.

Mechanisms	Model	Substance	Dose/Duration	Findings
Metabolic enzymes alteration and genetic polymorphism	SD rats [31]	RPM extract	6 g raw RPM/kg/bolus	Protein expression: CYP3A4, CYP2C19, CYP2E1, UGT1A1 and UGT1A8 ↓; ALT and AST ↑.
	SD rats [63]	RPM aqueous extract	40 g raw RPM/kg/3 weeks	CYP1A2 or CYP2E1 inhibitors + RPM: ALT and AST↑; moderate liver injury.
	Human (43 cases) [65]	RPM	NR	CYP1A2*1C frequency: 46.5%: RPM induced liver injury patients; 27.9%: healthy controls.
	Human (87 cases) [66]	RPM	4 weeks	HLA-B*35:01 allele: 45.4%: RPM induced liver injury patients; 2.7%: Han Chinese population.
Hepatocytes apoptosis	SD rats [69]	Emodin	1500 mg/kg/7 days	Emodin: ↑caspase-9, caspase-3, and Cyt c → mitochondrial apoptosis and liver injury
	L02 cells [70]	Emodin	50 μM	Emodin: ↑caspase-3 and ROS, ↓mitochondrial membrane potential, disrupting ATP synthesis → mitochondrial damage and hepatocyte apoptosis.
	HepaRG cells [71]	Emodin	20–80 μM	Emodin: cell cycle arrest and ROS generation → mitochondrial apoptosis → cell apoptosis.
Bile acids homeostasis disruption	SD rats [73]	Extracts of raw RPM (75% EtOH)	1 and 20g extract/kg/90 days	Protein expression of 3-hydroxy-3-methylglutaryl CoA reductase and CYP7A1 ↑ in a dose-dependent manner.
	SD rats [74]	RPM concentrated powder (1:10)	30 and 60g extract/kg/28 days	mRNA and protein expression of MRP2/*Mrp2*, MRP3/*Mrp3*, BSEP/*Bsep*, FXR/*Fxr*, CYP7A1/*Cyp7a1* ↑.
	SD rats [75]	Extracts of raw RPM (75% EtOH)	1 and 20 g extract/kg/ 3, 6, 7 weeks	HDCA, CA, TUDCA, and DCA in serum, TβMCA, TCA, CA, and βMCA in urine ↑ in a dose- and time-dependent manner; HDCA in serum and TβMCA in urine were identified as potential biomarkers for RPM induced liver injury; The mRNA expression of *Bsep* ↑ and *Ntcp* ↓ in liver.
	SD rats [76]	Extracts of raw and processed RPM (75% EtOH)	50 g extract/kg/42 days	GDCA in bile, as well as HDCA in serum, could be selected as potential biomarkers for RPM induced liver injury.
	C57BL/6J mice [77]	Extracts of processed RPM (60% EtOH)	1.275 and 3.825g extract/kg/7 days	Total bile acids↓ in liver and serum, unconjugated BAs ↑ in intestines; mRNA expression: *Nctp* and *Bsep*↑; protein expression of CYP7A1 ↓.
	Sandwich cultured rat hepatocytes [78]	Emodin, Physcion, Chrysophanol	1–50 μM	All compounds could alter bile acids disposition through direct ↓BA transporters as well as regulated expression of bile acids transporters and metabolic enzymes.
Inflammatory damage	SD rats [79]	Emodin	20, 40, 80 mg/kg	Emodin + lipopolysaccharide: ↑ proinflammatory cytokines (TNF-α, IL-1β and IL-6) → ALT and AST ↑.
	L02 cells [80]	Emodin	10.93, 54.09, 267.7 μM	Emodin: ↑ p-NF-κB and IL-6 → inflammatory damage.

NR: not reported, ↑: increase, ↓: decrease.

## Abbreviations

AUC	Area under the concentration-time curve
ADME	Absorption, distribution, metabolism and elimination
BSEP	Bile salt export pump
C_max_	Peak concentration
CL_int_	Intrinsic clearance
CYP	Cytochrome P450
Cyt c	Cytochrome c
EMG	Emodin-8-*O*-*β*-D-glucopyranoside
FXR	Farnesoid X receptor
GCDCA	Glycochenodeoxycholic acid
HLA	Human leukocyte antigen
HDCA	Hyodeoxycholic acid
K_a_	Absorption rate constant
LPS	Lipopolysaccharide
MRP	Multidrug resistance-associated protein
NTCP	Sodium taurocholate co-transporting polypeptide
P_app_	Apparent permeability coefficient
P_eff_	Effective intestinal permeability
RPM	Radix Polygoni Multiflori
SGLT1	Na^+^/glucose cotransporter
TSG	2,3,5,4′-tetrahydroxystilbene-2-*O*-*β*-D-glucopyranoside
T_max_	Time to maximum plasma concentration
TβMCA	Tauro-β-muricholic acid
UGT	UDP glycosyltransferase
